# The Multifaceted Regulation of Mitochondria in Ferroptosis

**DOI:** 10.3390/life11030222

**Published:** 2021-03-10

**Authors:** Hao Wu, Fengli Wang, Na Ta, Ting Zhang, Weihua Gao

**Affiliations:** 1 State Key Laboratory of Agricultural Microbiology, College of Veterinary Medicine, Huazhong Agricultural University, Wuhan 430070, China; tzh3058@webmail.hzau.edu.cn; 2 Institute of Reproductive Health, Tongji Medical College, Huazhong University of Science and Technology, Wuhan 430030, China; wangfengli@hust.edu.cn; 3 State Key Laboratory of Membrane Biology, Institute of Zoology, Chinese Academy of Sciences, Beijing 100101, China; tana@ioz.ac.cn; 4 State Key Laboratory of Agricultural Microbiology, College of Animal Science and Technology, Huazhong Agricultural University, Wuhan 430070, China; weuhuagao@webmail.hzau.edu.cn

**Keywords:** ferroptosis, mitochondria, cell death, iron, lipid peroxidation

## Abstract

Ferroptosis is characterized as a novel form of regulated cell death, which is initiated by the lethal accumulation of lipid peroxidation catalyzed by cellular labile free iron. This iron driven cell death sharply differs from other well characterized forms of regulated cell death at morphological, genetic and biochemical levels. Increasing research has elaborated a high relevance between dysregulated ferroptosis and the pathogenesis of degenerative diseases and organs injury in human patients. Additionally, targeted induction of ferroptosis is considered as a potentially therapeutic design for the clinical intervention of other therapy-resistant cancers. It is well understood that mitochondria, the cellular powerhouse, determine several types of regulated cell death. Recently, compromised mitochondrial morphology and functionalities have been primarily formulated in ferroptosis. Several mitochondria associated proteins and metabolic processes have been elaborated to fine-tune ferroptotic program. Herein, we critically review the recent advances in this booming field, with focus on summarizing the multifaceted mitochondrial regulation of ferroptosis and providing a perspective on the potential biochemical basis. Finally, we are attempting to shed light on an integrative view on the possibility of mitochondria- and ferroptosis-targeting therapeutics as novel treatment designs for the intervention of ferroptosis related diseases.

Regulated cell death (RCD, or programmed cell death, PCD) is a fundamental process essential for maintaining cell and tissue homeostasis in metazoans. RCD is tightly organized by signaling cascades and successively executed by a series of unique molecules. Based on the morphological, biochemical, and genetic characteristics, RCD is classified into distinct types, including apoptosis, necroptosis, pyroptosis, ferroptosis and other forms of RCD, according to the formulated guidelines of the Nomenclature Committee on Cell Death (NCCD) [1,2]. Among them, ferroptosis is a newly characterized form of non-apoptotic cell death driven by iron-dependent lipid peroxidation, due to the pharmacological or pathological perturbation of lipophilic antioxidant systems, especially the GSH (glutathione)-GPX4 (glutathione peroxidase 4) axis. During this decade, it has been gradually recognized that dysregulated ferroptosis is potentially implicated in the cell degeneration and tissue damage during the pathogenesis of several human diseases, especially the neurodegenerative diseases. In addition, targeted induction of ferroptosis provides promising antitumor therapies by triggering lethal cytotoxicity of cancer cells, which are potently resistant to other therapies. Therefore, ferroptosis has attracted increasing attention. In the current review, we will summarize the recent advances in this field and provide a critical perspective on the biochemical and genetic basis, with focus on formulating the multifaceted regulation of ferroptosis by mitochondria. Finally, we will also deliberate the possibility of ferroptosis- and mitochondria-targeting therapeutics for the clinical intervention of ferroptosis- or iron overload-associated human diseases.

## 1. A Brief Introduction of Ferroptosis

### 1.1. Pioneering Studies Related to Ferroptosis

Although ferroptosis was characterized and the term was coined by Dr. Brent R. Stockwell and his colleagues in 2012 [3], it is worth noting that earlier studies enlightened the pioneering understanding. In 1955, extracellular cystine was identified as the essential nutrient for cultured carcinoma cell HeLa and fibroblasts in vitro [4]. Cystine deprivation triggers cell death with a unique microscopic change [5]. This cell death is manifested by GSH exhaustion [6], and could be mitigated by lipophilic antioxidant α-tocopherol [6] or iron chelator DFO (deferoxamine) [7]. Additionally, extracellular glutamate overload induces cytotoxicity of neuronal cells by restraining cystine transport, leading to GSH exhaustion and accumulation of intracellular peroxides [7]. Although this glutamate excitotoxicity was originally referred to oxytosis [8], and alternative mechanisms were reported to be involved in [9,10], it is still unknown whether oxytosis and ferroptosis are similar or even exactly identical form of RCD [11]. These earlier studies thus established the preliminary understanding of cysteine/cystine deprivation, glutamate overload and GSH exhaustion in the process of iron and oxidation dependent cell death, which are now considered as the fundamental features of ferroptosis.

### 1.2. The Discovery of Ferroptosis

In 2003, by using a synthetic lethal compound high-throughput screening, Dr. Stockwell and the colleagues identified a novel antitumor compound named erastin, which could trigger lethal cytotoxicity of the engineered tumorigenic cells expressing mutant Ras oncogene, but not their isogenic normal cell counterparts [12]. A later synthetic lethal screening assay in 2008 identified two oncogenic-Ras-selective lethal compounds, RSL3 and RSL5, which trigger apoptosis independent cell death in human foreskin fibroblasts (BeJLR cells) [13]. Erastin and RSL induced cell death differs sharply from other well characterized forms of RCD. Morphologically, the ballooning phenotype, mainly characterized by the formation of a clear rounded cell consisting of empty cytosol, can be microscopically observed during ferroptosis, but not other types of cell death [14]. Biochemically, neither cytochrome c release nor PARP1/Caspase 3 cleavage takes place upon erastin or RSL3 challenge [15]. Additionally, the pro-apoptotic proteins Bax and Bak, as well as the pro-necroptotic proteins RIP1 and RIP3, are dispensable for erastin or RSL3 induced cell death [16]. Chemical inhibition of apoptosis or necroptosis by the corresponding inhibitor fails to mitigate this cell death. In striking contrast, the iron chelators and ROS (reactive oxygen species) scavengers could almost completely block this lethal cytotoxicity, suggesting an iron- and oxidation-dependency of this non-apoptotic cell death.

### 1.3. The Core Regulatory Circuit of Ferroptosis

**Figure 1:**
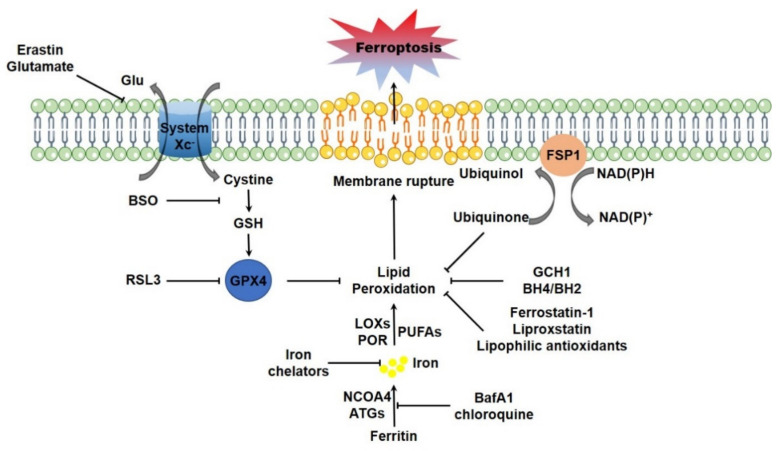
The main regulatory circuit of ferroptosis. System X_C_^-^ mediated cystine uptake-GSH synthesis-GPX4, FSP1-CoQ-NADPH and GCH1-BH4/BH2 are major pathways for suppressing lipid peroxidation. Erastin, BSO and RSL3 are typically targeting to system X_C_^-^, GSH synthesis, and GPX4, respectively, to disrupt these endogenous lipophilic antioxidant systems and elevate lipid peroxidation. The LOXs and POR could enzymatically catalyze the formation of lipid peroxides, while cellular free iron catalyzes lipid peroxidation via Fenton rection or via the LOXs or POR. Autophagic ferritin degradation (ferritinophagy) could motivate cellular iron and facilitate lipid peroxidation. Iron chelators, as well as the autophagy inhibitors including BafA1 and chloroquine, could suppress lipid peroxidation and counteract ferroptosis by decreasing cellular free iron. Furthermore, the lipophilic antioxidants could directly trap lipophilic radical to halt the propagation of lipid peroxidation and suppress ferroptosis.The lethal outburst of lipid peroxides ultimately drives ferroptosis. Abbreviations: ATGs, autophagy genes; BafA1, bafilomycin A1; BH4/BH2, tetrahydrobiopterin/dihydrobiopterin; BSO, buthionine sulfoximine; FSP1, ferroptosis suppressor protein 1; GCH1, GTP cyclohydrolase 1; Glu: glutamate; GPX4, glutathione peroxidase 4; GSH, glutathione; LOXs, lipoxygenases; NADPH, β-nicotinamide adenine dinucleotide 2′-phosphate, reduced; NCOA4, nuclear receptor coactivator 4; POR, cytochrome P450 oxidoreductase; PUFAs, polyunsaturated fatty acids.

Erastin binds to the cystine/glutamate antiporter (system X_C_^-^, a member of the heteromeric amino acid transporter family, typically exchanges the extracellular cystine and intracellular glutamate, composed of a 12-pass transmembrane component SLC7A11 and a 1-pass transmembrane regulatory component SLC3A2 [17]), interferes the cystine uptake and thus disrupts the biosynthesis of GSH, the γ-L-glutamyl-L-cysteinyl-glycine tripeptide as a key determinant of cellular redox homeostasis [3]. RSL3 fails to impinge on the cystine uptake and GSH biosynthesis, but alternatively targets to GPX4 directly and restrains its enzymatic activity [18]. GPX4 is a member of GPXs family, which catalyzes the reduction of hydrogen peroxide and organic hydroperoxides by using GSH as the essential cofactor. It was reported that GPX4 is the unique antioxidant enzyme for the ability to directly reduce phospholipid hydroperoxides and oxidized lipoproteins to their respective lipid-alcohol within cellular bio-membranes [19]. Therefore, targeted deletion of *GPX4* elevates lipid peroxidation and thus triggers ferroptotic cell death in cultured mammalian cells [20]. Cystine deprivation or chemical inhibition of GSH biosynthesis leading to GPX4 inactivation also initiates ferroptotic cell death [18,21,22,23]. Moreover, genetic ablation of *GPX4* leads to early embryonic lethality [24,25], while inducible deletion of *GPX4* results in acute renal failure and early death in mice [20]. By using T lymphocytes specific *GPX4* knockout mice as a model, it was reported that GPX4 is vital for the homeostatic survival of CD8^+^ T cells in the periphery (spleen, peripheral lymph node and mesenteric lymph node), as well as for the expansion of both CD4^+^ and CD8^+^ T cells upon T cell receptor triggering in response to infection through preventing lipid peroxidation and counteracting ferroptosis [26]. Additionally, inducible depletion of *GPX4* specifically in neuron causes rapid paralysis, muscle atrophy and early death. A ferroptotic degeneration of spinal cord motor neurons is observed in these mutant animals [27,28]. Other studies using tissue specific *GPX4* knockout mice as models therewith demonstrated the critical importance of GPX4-GSH system in maintenance of hepatocellular homeostasis and normal liver function [29], survival of endothelial cells [30], as well as the maturation of photoreceptor cells [31]. Collaboratively, these studies thus highlight the core regulatory circuit composed of system X_C_^-^ mediated cystine uptake-GSH biosynthesis-GPX4-lipid peroxidation in navigating ferroptotic program. More recently, two independent groups identified a novel regulatory circuit parallel to GPX4-GSH system in ferroptosis regulation. Flavoprotein AIFM2 (apoptosis-inducing factor mitochondria-associated 2), which was initially identified as a pro-apoptotic and P53-responsive protein associated with mitochondrial outer membrane [32,33], was recently characterized as a novel ferroptosis modulator. Myristoylation of AIFM2 leads to a plasma membrane translocation, where AIFM2 serves as a NADPH (β-nicotinamide adenine dinucleotide 2′-phosphate, reduced) dependent oxidoreductase to mediate the reduction of ubiquinone (also known as coenzyme Q10, CoQ10), trap lipid peroxyl radicals and thus suppress lipid peroxidation. AIFM2 is thus renamed FSP1 (ferroptosis suppressor protein 1) [34,35]. Unlike the embryonic lethality of *GPX4* knockout in mice, *FSP1* knockout mice are produced normally and are without any overt abnormal phenotypes up to 1 year old [36]. This is possibly due to the compensatory detoxification of lipid peroxidation by GPX4. Additionally, it was just recently reported GCH1 (GTP cyclohydrolase-1), the rate-limiting enzyme catalyzing the biosynthesis of BH4 (tetrahydrobiopterin) and BH2 (dihydrobiopterin), as another highly potent endogenous inhibitory protein for ferroptosis. Specifically, *GCH1* expression leads to the enhanced synthesis of BH4 and BH2, which facilitate lipid remodeling by selectively preventing the depletion of phospholipids with two polyunsaturated fatty acyl tails, and thus antagonize ferroptosis, without any overt impact on other ferroptosis regulators [37] (Figure 1).

## 2. Lipid Peroxidation Driving Ferroptosis

Accumulation of lipid peroxides directly executes ferroptotic cell death. Although erastin and RSL3 do elevate the cytosolic superoxide, but not limited to the lipid peroxides, it seems that the cytosolic superoxide dose not contribute to ferroptosis as the compounds elevating cytosolic superoxide fail to trigger ferroptosis [38].

**Figure 2:**
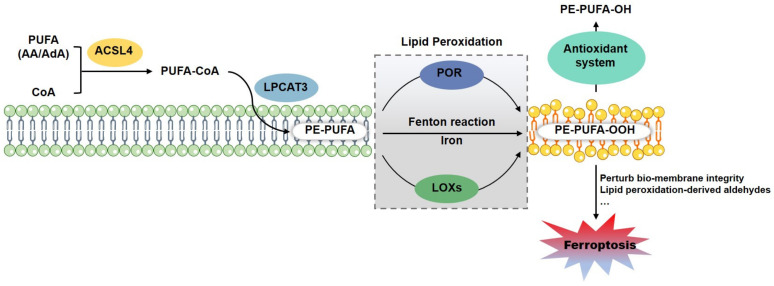
Lipid Peroxidation drives ferroptotic cell death. ACSL4 and LPCAT3 motivate and esterify the PUFA (AA and AdA) into PE for the next peroxidation, which is enzymatically catalyzed by LOXs or POR, or non-enzymatically mediated by cellular free iron via Fenton reaction. Lipid peroxides could be antagonized by lipophilic antioxidant system (including GPX4-GSH, FSP1-CoQ-NADPH and GCH1-BH4/BH2 axises). The lethal accumulation of lipid peroxides could drive ferroptotic cell death directly through perturbing the integrity of bio-membrane, or indirectly via the peroxidation-derived aldehydes. Abbreviations: AA, arachidonic acid; ACSL4, acyl-CoA synthetase long-chain family member 4; AdA, adrenaline; LOXs, lipoxygenases; LPCAT3, lysophosphatidylcholine acyltransferase 3; PE, phosphatidylethanolamine; POR, cytochrome P450 oxidoreductase; PUFAs, polyunsaturated fatty acids.

Lipidomic studies revealed that PUFAs (polyunsaturated fatty acids)-containing PLs (phospholipids), especially PEs (phosphatidylethanolamines), are the most susceptible lipids to peroxidation at the bis-allylic position during ferroptosis [39,40]. Oxygenated PEs are accumulated in *GPX4* inactivated cells and in *GPX4* ablated kidney tissue. It is worth noting that free PUFAs, such as AA (arachidonic acid) and AdA (adrenaline), must be esterified into PLs for the peroxidation. ACSL4 (Acyl-CoA synthetase long-chain family member 4) and LPCAT3 (lysophosphatidylcholine acyltransferase 3) have been identified for this PUFAs incorporation and lipid remodeling during ferroptosis [41,42] (Figure 2). Genetic or pharmacological inhibition of ACSL4 leads to a substantial ferroptotic insensitivity [40,41]. Unlike to the PUFAs, exogenous MUFAs (monounsaturated fatty acids) potently restrain ferroptotic cell death by decreasing oxidizable PUFAs-containing PLs and thus remodeling membrane lipidome [43].

### 2.1. Lipid Peroxidation Enzymatically Catalyzed by LOXs and POR

Oxidation of PUFA-PLs could be enzymatically catalyzed by LOXs (lipoxygenases), COXs (cyclooxygenases), and CYPs (cytochrome P450), as well as non-enzymatically catalyzed by free iron via Fenton reaction [44] (Figure 2). LOXs, the non-heme iron-containing dioxygenases encoded by the *ALOXs* genes, are identified as the most critical molecules for lipid peroxidation. Transfection of the siRNA pools targeting all *ALOXs* genes or general pharmacological inhibition of LOXs strikingly prevents erastin induced lipid peroxidation and ferroptosis [39]. Among them, it is still elusive whether any unique LOX functions dominantly or they catalyze lipid peroxidation redundantly, due to the different expression patterns in different tissues and cells. The siRNA pools targeting *ALOX15B* and *ALOXE3* could efficiently prevent the lethal cytotoxicity of erastin in BJeLR and HT-1080 cells [39]. Another study showed that 12/15-LOX is indispensable for ferroptosis in *GPX4* knockout or inactivated cells [28]. PEBP1 (PE-binding protein 1), which was previously identified as the RKIP1 (Raf1 kinase inhibitory protein) to interact and inhibit the Raf1 kinase cascade, is a master regulatory molecule for the LOXs, especially 15-LOX. PEBP1 modulates the substrate specificity of 15-LOX to PUFA-PEs, leading to generation of HpETE-PEs [45]. However, only pharmacological inhibition of LOXs by the 12/15-LOX inhibitor has been evidenced to efficiently protect *GPX4* deficient cells from death [28], while specific ablation of *ALOX15* gene does not rescue homozygous knock-in mice expressing catalytically inactivated GPX4 mutant Sec46Ala from embryonic lethality [46]. It was reported that the ferroptosis inhibitor liproxstatin-1 could suppress the production of pro-ferroptotic oxygenated PEs by inhibiting the enzymatic activity of 15-LOX [40]. However, other study reported that liproxstatin-1 and ferrostatin-1 perform poor inhibition directly on 15-LOX, but alternatively present as radical-trapping antioxidants to suppress lipid peroxidation [47]. These evidences thus indicate that it is reasonable additional molecules may exist to catalyze the lipid peroxidation bypass LOXs [48].

POR-CYP system is typically composed of a POR (cytochrome P450 oxidoreductase) and a CYP (cytochrome P450) isoenzyme that contains a protoporphyrin IX heme-iron prosthetic group [49]. It is known that POR-CYP plays key roles in maintaining cellular redox homeostasis and detoxification of xenobiotic chemicals. Just recently, by using genome-wide CRISPR-Cas9 screening, Zou and colleagues reported POR as a critical modulator of ferroptotic cell death. Redox lipidomics assay showed that POR depletion significantly reduces both hydroperoxyl- and hydroxyl-PUFA-PEs, suggesting that POR facilitates lipid peroxidation [50] (Figure 2). CYP accepts electrons from POR, and simultaneously catalyzes the peroxidation of PUFA lipids, while POR facilitates lipid peroxidation by accelerating the switch between Fe^2+^ and Fe^3+^ in the heme prosthetic group of CYP [51,52]. However, the CRISPR-Cas9 screening was not able to nominate any specific CYP as the major POR partner in catalyzing lipid peroxidation and facilitating ferroptosis [50]. Just recently, Yan and colleagues reported that POR-CYP interactions are dispensable to POR-mediated ferroptosis [53].

In addition, there is no direct evidence presenting a causal link between COXs mediated lipid peroxidation and ferroptosis execution. COX-2, encoded by *Ptgs2* gene, is transcriptionally upregulated during ferroptosis, which is now considered as one of the major hallmarks of ferroptosis. However, chemical inhibition of COX-2 fails to affect ferroptotic cell death [18].

### 2.2. Lipid Peroxidation Non-Enzymatically Catalyzed by Iron

The non-enzymatic lipid peroxidation refers to Fenton chemistry, which is accomplished by the cellular labile free ferrous iron. The cellular iron is always ligated by heme or ISC (iron-sulfur cluster) in the heme- or ISC-containing proteins, respectively, or tightly chelated by ferritin [54]. There is a small portion of cellular ferrous iron (labile iron pool) that is loosely ligated and could catalyze lipid peroxidation via Fenton chemistry [55,56]. In cells, the labile iron pool is highly dynamic. The iron-containing ferritin could be selectively degraded via proteasome dependent manner [57] or autophagy dependent manner (which is termed ferritinophagy) [58,59]. Free iron is thus released for the cellular utilization. It was reported that ferritinophagy is initiated and free iron is thus elevated during ferroptosis. Knockdown of core autophagy components including *ATG3*, *ATG13*, *ATG5* and *ATG7*, or the specific ferritinophagy receptor *NCOA4*, or pharmacologically inhibition of autophagy by the lysosomal inhibitor BafA1 or chloroquine, significantly mitigates ferroptotic cell death [60,61] (Figure 1). Likewise, activation of HO-1 (heme oxygenase-1), which enhances heme catabolism and iron release, is expectable to accelerate ferroptotic program. The specific HO-1 inhibitor and genetic ablation of *HO-1* could markedly combat ferroptosis [62,63,64]. However, the other study raised an opposite argument that HO-1 protects cells from ferroptosis [65]. The redox active ferrous iron mediates the initiation step for the generation of reactive hydroxyl radical in Fenton reaction [66]. Additionally, the switch between Fe^2+^ and Fe^3+^ redox states also supports the catalytic reaction of LOXs and CYPs for the corresponding lipid peroxidation [67,68].

### 2.3. Lipid Peroxidation Driving Ferroptotic Cell Death

It was well understood that certain type of lipid peroxidation could drive certain form of RCD, including apoptosis [69] and pyroptosis [70,71]. However, it may be more complicated for lipid peroxidation to drive ferroptotic cell death. The lipid peroxidation may kill cells directly by damaging the bio-membranes or indirectly through the metabolic derivatives [72]. On one hand, lipid peroxidation perturbs the integrity of the bio-membrane bilayer structure. More specifically, the oxidized phospholipids confined in bio-membrane could change the bio-membrane fluidity, bio-membrane permeability, as well as bio-membrane associated signaling and metabolic processes leading to cell death [73]. The lipid peroxidation alters the local shape and curvature of lipid membranes, promotes accessibility to oxidants, which expedites membrane destruction and triggers ultimate cell death [74]. Additionally, phospholipids peroxidation would generate a variety of oxidized phospholipids containing a shortened fatty acyl chain by the rearrangement of alkoxyl radicals. These short-chain phospholipids perform a detergent-like activity [75] and secondarily damage bio-membrane [76,77]. On the other hand, lipid peroxidation-derived aldehydes including 4-HNE (4-hydroxy-2-nonenal), 4-HHE (4-hydroxy-2-hexenal), and MDA (malondialdehyde), could diffuse and react with proteins, DNA, and phospholipid molecules to generate a variety of intra-molecular and inter-molecular covalent adducts [44,78]. Specifically, MDA could covalently bind multiple proteins including enzymatic proteins, carrier proteins, cytoskeletal proteins, mitochondrial proteins as well as antioxidant proteins [79]. A recent study by using a quantitative chemoproteomic method to profile protein carbonylation showed that more than 400 proteins are carbonylated during ferroptosis, which is potently driven by these reactive aldehydes. Among them, several ferroptosis associated molecules have been identified [80]. In addition, these aldehydes could act as signaling molecules to drive cell death [81]. Nevertheless, the exact mechanism for which lipid peroxidation drives ferroptotic cell death is to be investigated [72] (Figure 2).

## 3. Disrupted Mitochondrial Integrity during Ferroptosis

The cellular powerhouse mitochondria control a set of critical biological processes, including energy production via electron transport coupled with oxidative phosphorylation, fatty acid β-oxidation, TCA (tricarboxylic acid) cycle, iron metabolism, as well as calcium homeostasis. It is well understood that mitochondria also function as a signaling hub to orchestrate intracellular or extracellular clues and communicate to other cellular compartments [82,83]. In addition to such diverse functions, mitochondria also determine several types of cell death, including apoptosis [84,85], pyroptosis [86], and necroptosis [87].

Mitochondria are highly dynamic organelle with constant fusion, fission and turnover. The mitochondrial quality and quantity are systematically coordinated by the mitochondrial biogenesis and mitochondrial clearance [88]. The mitochondrial architecture is mainly determined by the dynamin-related GTPases MFN1/MFN2 (which mediate fusion of mitochondrial outer membrane) and OPA1 (which mediates fusion of mitochondrial inner membrane) driven fusion, as well as by the cytosolic protein DRP1 and its mitochondrially localized receptors driven fission [89,90]. It has been well understood that mitochondrial dynamics are substantial for compensating mitochondrial damage through fusion and eliminating mitochondria with unrecoverable damage through fission. Increasing number of studies have elaborated a tight association between disturbed mitochondrial dynamics and the occurrence or development of diverse human diseases [91,92]. Mitochondrial biogenesis is composed of multiple steps including replication, transcription and translation of mtDNA, transcription and translation of nuclear-encoded proteins, biosynthesis and transport of mitochondria associated lipid, which are mainly orchestrated by several transcriptional factors, including PGC-1α, NFR1, and TFAM [93,94]. Mitochondrial clearance is mainly achieved by mitophagy, which refers to a highly selective recognition and removal of mitochondria by the autophagosome-lysosome machinery [95,96]. To data, PINK1-Parkin dependent mitophagy and receptors mediated mitophagy have been dissected in mammalian cells [97].

### 3.1. The Engagement of Mitochondria in Iron Overload Associated Cell Damage

Trace elemental iron is one of the essential nutrients for cell growth. However, iron overload, one of the most substantial causes and typical hallmarks of ferroptosis, facilitates mitochondrial damage at distinct levels [98,99]. Iron overload leads to the abnormal mitochondrial morphology, resulting in a fragmented architecture in several types of cultured mammalian cells [100,101]. Excess iron supplementation also compromises mitochondrial functionalities by restricting mitochondrial oxidative phosphorylation and antioxidant response [102]. The mtDNA is vulnerable to iron deposition. It was reported that double strand breaks of mtDNA are observed in iron-exposed mitochondria. Iron overload also leads to progressive loss of intact mtDNA, as well as reduced mtDNA transcription and decreased expression of respiratory chain subunits encoded by mitochondrial genome [103,104,105,106]. Conversely, preserving mitochondrial architecture and functionalities by utilizing distinct approaches was evidenced to protect cells from iron toxicity in multiple models. Specifically, inhibition of DRP1 mediated mitochondrial fragmentation prevents ferric ammonium citrate-induced neuronal cell loss [107]. Injection of the mitochondrially targeting antioxidant mito-TEMPOL [108] could alleviate hepatotoxicity and lower hepatocytic death induced by excess dietary iron [109]. These studies thus enlightened a causal role of mitochondrial dysfunction in iron overload mediated cell damage, which is now considered to highly relate to ferroptosis.

### 3.2. Disrupted Mitochondrial Morphology in Ferroptosis

As previous study demonstrated a potential binding to the multifunctional mitochondrial proteins VDAC2/3 (voltage-dependent anion channel 2/3) of erastin [15], a plausible involvement of mitochondria in ferroptosis regulation was hypothesized. Upon exposure of erastin, shrunken mitochondria appear smaller with increased membrane density in BJeLR cells as imaged by the transmission electronic microscope [3]. A similar phenotype was observed in HT-22, a mouse hippocampal neuronal cell Line. Erastin induces mitochondrial fragmentation and accumulation around the nucleus as shown by the MitoTracker DeepRed staining [110]. Similarly, exposure to BSO (buthionine sulfoximine), the chemical inhibitor of γ-GCS (γ-glutamylcysteine synthetase) essential for GSH biosynthesis, leads to lamellar and tubular structured mitochondria with diminished cristae [20]. *GPX4* ablated cells contain swollen mitochondria with a reduced number of cristae and a lamellar architecture. Furthermore, exposure of RSL3 leads to a rupture of mitochondrial outer membrane in Pfa1 cells in a time-dependent manner [20]. Currently, abnormal mitochondrial architecture including mitochondrial fragmentation, shrunken mitochondria and rupture of mitochondrial outer membrane, as well as vanished mitochondrial cristae, is regarded as the typical morphological characteristic of ferroptotic cell death [111].

### 3.3. Mitochondrial ROS Burst in Ferroptosis

No increase in mitochondrial ROS was observed in erastin treated HT-1080 cells as evidenced by mitoSOX (the specific mitochondrial ROS probe) staining, although erastin significantly elevates both cytosolic and lipid ROS [3]. However, Carsten Culmsee and colleagues demonstrated a substantial increase of mitochondrial ROS in erastin challenged HT-22 and MEF cells [110]. This group also reported that exposure of RSL3 similarly triggers a burst of mitochondrial ROS in MEF and HT-22 cells [112]. This discrepancy may be due to the different cells used or different exposure time. Mitochondria-targeted ROS scavenger MitoQ (mitoquinone) can protect neuronal cells from RSL3 induced ferroptosis [112]. Compared to a more broadly distributed antioxidant, the specifically mitochondria-targeted nitroxide performs a more potent effect on protecting cells from ferroptotic cell death [113]. These studies thus collaboratively indicate the possibility of an engagement of mitochondrial ROS in navigating ferroptotic program.

### 3.4. Altered Mitochondrial Membrane Potential in Ferroptosis

The mitochondrial membrane potential (ΔΨm) generated by proton pumps is essential for ATP production by coupling with oxidative phosphorylation [114]. It was reported by Dr. Xuejun Jiang’s lab that diverse ferroptosis inducers, including cystine starvation, amino acid-free medium plus full serum (induces ferroptosis that was reported in [115]), erastin and glutamate can all induce mitochondrial hyper-polarization by using TMRE (tetramethylrhodamine ethyl ester) staining, while the mitochondrial uncoupler CCCP could disrupt mitochondrial membrane potential and almost completely block cystine starvation induced lipid ROS accumulation and ferroptosis [116]. Furthermore, previous study demonstrated a potential binding to VDAC2 and VDAC3 of erastin [15], while RSL3 exposure leads to the carbonylation of VDAC2 [80]. It is reasonable that the direct binding to VDACs of erastin or the carbonylation of VDAC2 by RSL3 may result in the conformational change of VDACs, and thus lead to mitochondrial hyper-polarization [117,118], as VDACs serve as a gatekeeper to facilitate transmembrane exchange of ions and metabolites [117,119,120]. This is confirmed by a NADH oxidation assay, and erastin treatment yields a decrease in the rate of NADH oxidation, suggesting a decreased permeability of mitochondria to NADH [15]. Depolarization of mitochondria could restrain cystine starvation induced, but not *GPX4* knockout induced ferroptosis [20]. This is consistent with the recent study from Dr. Xuejun Jiang’s lab. ETC inhibitors including the mitochondrial uncoupler CCCP sharply suppress lipid peroxidation and ferroptosis induced by erastin or cystine deprivation [116]. However, these ETC inhibitors fail to impinge on lipid ROS accumulation and ferroptosis upon GPX4 inhibition, neither induced by RSL3 nor by *GPX4* knockout [116]. This is possible that mitochondria function upstream of GPX4 to result in an exhaustion of GSH. In striking contrast, the studies presented by Carsten Culmsee group reported a profound loss of mitochondrial membrane potential upon the exposure of glutamate, erastin or RSL3 in MEF and HT-22 cells by using the identical TMRE staining [110,112].

### 3.5. Elevated Mitochondrial Lipid Peroxidation in Ferroptosis

*GPX4* knockout kidneys show an accumulation of oxidized cardiolipin [20]. Examination of subcellular localization of the lipid ROS by confocal imaging showed that the lipid peroxidation first appears in a distribution significantly co-localized with mitochondria, then trans-localizes with the plasma membrane at later time points [116]. This is consistent with the pattern of doxorubicin-induced myocardial ferroptosis. Fang et al. showed that lipid peroxidation is specifically increased inside the mitochondria, but not in the cytoplasm by utilizing a mitochondria fractionation assay. Moreover, mitochondria-targeted antioxidant mito-TEMPOL [108], but not the non-mitochondria targeted antioxidant TEMPO, could significantly abolish doxorubicin induced lipid peroxidation and cardiac ferroptosis, thus highlighting a critical importance of mitochondrial lipid peroxidation in navigating ferroptosis [64]. However, other report suggested that ferroptosis is initiated by extra-mitochondrial lipid peroxidation [20].

Altogether, the current research demonstrates disrupted mitochondrial integrity, including abnormal mitochondrial architecture, burst of mitochondrial ROS, alteration of mitochondrial membrane potential, as well as elevated mitochondrial lipid peroxidation during ferroptosis. Overall, mitochondrial compromise plays a potent role in class I ferroptosis, which is dependent on GSH exhaustion, but not in class II ferroptosis, which is dependent on genetic ablation or pharmacological inhibition of GPX4. Opposite conclusions proposed from different studies may be due to the different cell lines or different methodologies utilized. Specifically, the cell viability assays based on mitochondrial respiration maybe not suitable to evaluate the role of mitochondria in ferroptosis, as manipulation of mitochondrial function may impact the outcome of the cell viability assays [116].

## 4. Mitochondria Manipulating Ferroptosis

**Figure 3:**
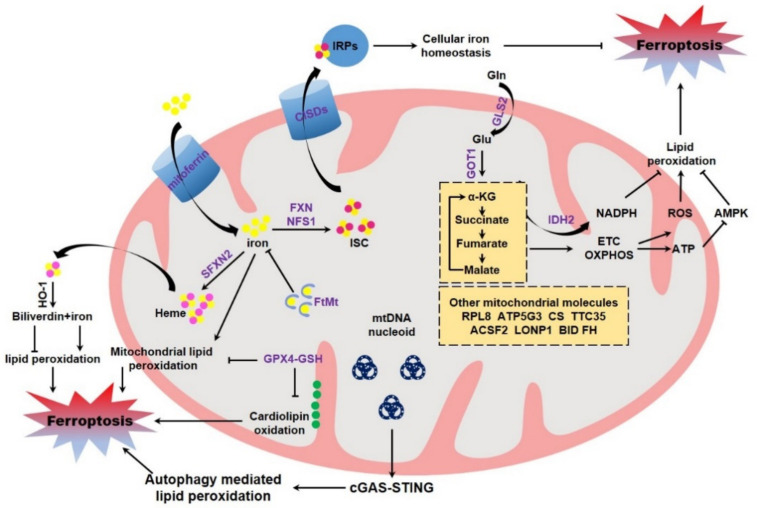
Mitochondrial regulation of ferroptosis. Mitochondria modulate ferroptotic cell death through multifaceted mechanisms. As the major compartment for cellular iron metabolism, mitochondria determine the ISC assembly and heme bio-synthesis, and thus dominate the enzymatic activities of numerous proteins containing ISC or heme. Mitochondria also govern cytosolic iron via ISC/IRPs or heme/HO-1 axis. The related mitochondrial proteins including mitoferrin, FXN, NFS1, SFXN2, FtMt, and CISDs thus modulate cellular iron metabolism and ferroptotic sensitivity. The mitochondrial glutaminolysis-TCA-ETC axis is other major pathway to determine ferroptotic cell death, during which the production of ROS, NADPH, and ATP regulates ferroptosis through distinct mechanisms. Mitochondrial GPX4/GSH also determines ferroptosis, which may be partially via modulating cardiolipin oxidation. mtDNA stress could activate the cGAS-STING pathway to facilitate autophagy mediated lipid peroxidation. Moreover, other mitochondria associate molecules also participate in ferroptosis regulation. Abbreviations: ACSF2, acyl-CoA synthetase family member 2; AMPK, adenosine 5′-monophosphate (AMP)-activated protein kinase; ATP, adenosine triphosphate; ATP5G3, ATP synthase F0 complex subunit C3; BID, BH3-interacting domain death agonist; cGAS, cyclic GMP-AMP synthase; CISDs, CDGSH iron sulfur domain; CS, citrate synthase; ETC, electron transport chain; FH, fumarate hydratase; FtMt, mitochondrial ferritin; FXN, Frataxin; Gln, glutamine; GLS2, glutaminase 2; Glu, glutamate; GOT1, glutamic-oxaloacetic transaminase 1; GPX4, glutathione peroxidase 4; GSH, glutathione; HO-1, heme oxygenase-1; IDH2, isocitrate dehydrogenase 2; IRPs, iron regulatory protein; ISC, iron-sulfur cluster; α-KG, α-ketoglutarate; LONP1, Lon peptidase 1; mtDNA, mitochondrial DNA; NADPH, β-nicotinamide adenine dinucleotide 2′-phosphate, reduced; NFS1, NFS1 cysteine desulfurase; ROS, reactive oxygen species; RPL8, ribosomal protein L8; SFXN2, sideroflexin 2; STING, stimulator of interferon genes; TTC35, tetratricopeptide repeat domain 35.

The direct evidence to evaluate the potential role of mitochondria in modulating ferroptosis originates from the studies using mitochondria deficient cells. Gao et al. constructed the Parkin over-expressing HT-1080, and then CCCP was supplemented to remove mitochondria by continuous activation of PINK-Parkin dependent mitophagy [121,122]. By using these cells, the authors found that cells deficient in mitochondria are resistant to cystine deprivation or erastin exposure induced lipid peroxidation and ferroptosis, but remain sensitive to RSL3 induced lipid peroxidation and ferroptosis [116]. However, an opposite conclusion was drawn by Gaschler and colleagues. These authors reported that mitochondria are dispensable for ferroptosis execution by utilizing the identical methodology [123]. Mitochondria contain their own DNA (mtDNA) within the mitochondrial matrix. mtDNA is circular, double stranded, maternally inherited, and composed of 16,569 bp to encode 13 polypeptides of electron transport chain required for ATP production, as well as 22 tRNAs (transfer RNAs) and 2 rRNAs (ribosomal RNAs) [124,125]. It was reported that the mtDNA-depleted cells (termed ρ0 cells) generated from a long-term culture with ethidium bromide display an equivalent sensitivity to ferroptosis when compared to the parental cells [3]. Other study found that ρ0 cells are more sensitive to hydrogen peroxide-induced cell death through accelerating lipid peroxidation [126]. However, it is still unknown whether ferroptosis is involved in this cell death. Recently, Li and colleagues reported that zalcitabine, an antiviral drug for human immunodeficiency virus infection, triggers TFAM degradation dependent ferroptotic cell death of pancreatic cancer cells. Specifically, the degradation of TFAM triggers mtDNA stress, mtDNA release to the cytosol, and ultimate activation of the cGAS-STING pathway, the central cellular cytosolic double-stranded DNA sensor, allowing innate immune to respond to infections, inflammation, and cancer. STING mediated autophagy contributes to zalcitabine-induced ferroptosis by mediating lipid peroxidation [127] (Figure 3). However, it is unclear whether the typical ferroptosis inducers, including erastin, RSL3, *GPX4* knockout and cystine deprivation, could trigger the similar mtDNA stress.

### 4.1. Mitochondrial GPX4 Modulating Ferroptosis

Ferroptosis is dictated by multiple proteins and cellular metabolisms, of which some are tightly associated with mitochondria (Figure 3). GSH reduction and GPX4 inactivation are canonical hallmarks of ferroptosis. Mitochondria possess abundant GSH, with about 10–15% of total GSH [128], although GSH is not de novo synthesized inside of mitochondria [129]. Multiple mitochondria associated carrier proteins have been identified to mediate GSH importing into mitochondria for the maintenance of mitochondrial redox homeostasis. These include 2-oxoglutarate carrier (or SLC25A11), dicarboxylate carrier (or SLC25A10) and tricarboxylate carrier (or SLC25A1) [130,131]. Mitochondrial GSH modulates apoptotic cell death through remodeling mitochondrial membrane and restraining cardiolipin oxidation, which is postulated to facilitate cytochrome c release [132]. GPX4 is partially localized in the intermembrane space of mitochondria [133,134]. *GPX4* knockout is embryonic lethal, while *GPX4*^+/−^ heterozygous cells and the heterozygous mice are markedly sensitive to oxidative stress, as compared to the wild-type cells and the control littermates, respectively [135]. By using *GPX4* transgenic mice as the model, it was evidenced that GPX4 could preserve mitochondrial ATP production and maintain mitochondrial membrane potential under oxidative stress [136]. Additionally, independent studies evidenced that specific expression of mitochondrial *GPX4* mitigates lipid peroxidation and counteracts cell death. More specifically, mitochondrial GPX4 protects cells from oxidized low-density lipoprotein and cholesterol hydroperoxide [137,138], which may potently trigger ferroptotic cell death. The direct evidence originates from recent studies. The chemotherapeutic agent doxorubicin is commonly used to treat breast cancer, leukemia, and many other types of malignancies [139]. However, the clinical application is limited due to its cardiotoxicities, including irreversible degenerative cardiomyopathy and congestive heart failure [140]. It was reported that doxorubicin triggers a ferroptotic cardiomyopathy [64]. Over-expression of both cytosolic *GPX4* and the mitochondrial *GPX4* could equally suppress doxorubicin induced accumulation of lipid peroxidation, and thus protect against doxorubicin-induced ferroptotic cardiomyopathy [141]. This study thus shed light on a critical importance of mitochondrial GPX4 in antagonizing ferroptosis (Figure 3).

### 4.2. Mitochondrial Iron Modulating Ferroptosis

Mitochondria function as the center for cellular iron metabolism. Receptor-mediated endocytosis is the major mechanism for cellular iron uptake. Specifically, extracellular transferrin-bound iron is absorbed by the membrane associated transferrin receptor [142]. Alternatively, cells could also absorb iron directly via DMT1 (divalent metal transporter 1) with the assistance of cell surface ferrireductases DCYTB (duodenal cytochrome b) [143,144]. A large portion of cellular iron is safely sequestered by ferritin, while other is imported into mitochondria by the mitochondrial iron transporter mitoferrin1 in erythroblasts [145] and mitoferrin2 in non-erythroid cells [146]. Mitochondrial iron is stored by FtMt (mitochondrial ferritin) inside the mitochondrial matrix [147] or utilized for the biosynthesis of heme and ISC, two pivotal iron containing molecules essential for numerous heme- and ISC-containing proteins, respectively [148,149]. The excess cytosolic iron could be exported by ferrous iron exporter FPN1 (ferroportin 1), which is precisely controlled by the peptide hormone hepcidin [150]. The cellular iron homeostasis and metabolism is under strict control, mainly by the IRPs (iron regulatory proteins)/IREs (iron-responsive elements) system. During iron deficiency, IRPs (including IRP1 and IRP2) are activated to bind to the conserved stem-loop IREs in UTRs (untranslated regions) of target mRNAs, which mostly encode proteins associated with iron metabolism. The IRPs binding thus determines the fates of target mRNAs and leads to increased iron absorption, as well as decreased iron export, iron utilization and iron storage, thereby maintaining appropriate intracellular iron concentrations [151]. It was not unexpectedly that IRPs are crucial in determining the ferroptotic sensitivity. Independent studies using shRNA screening assay suggested that knockdown of both *IRPs* leads to an overt resistance to ferroptosis [3,60], which is possibly due to the reduced iron level in *IRPs* depleted cells. Iron overload, as mentioned above, could facilitate ferroptosis both in vivo and in vitro by catalyzing lipid peroxidation [152]. Immuno-depleted transferrin in serum or knockdown *TFRC* (transferrin receptor) protects cultured cells from ferroptotic cell death [115]. Additionally, mitochondria are iron overloaded in multiple ferroptosis models [64,153,154], although the underlying mechanism is unclear. It is reasonable that iron accumulation in mitochondria would lead to mitochondrial dysfunction, including disrupted mitochondrial morphology and compromised mitochondrial functionalities as mentioned above. Additionally, mitochondrial iron deposition also catalyzes lipid peroxidation in mitochondrial membrane and triggers ferroptosis. Therefore, it is not surprised that expression of *FtMt* significantly protects cells from ferroptosis by safely sequestering iron within mitochondria. Moreover, transgenic expression of *FtMt* protects the survival of fruit fly drosophila feeding an erastin containing diet [155,156], highlighting a fundamental role of mitochondrial iron in determining the ferroptotic sensitivity (Figure 3).

### 4.3. Mitochondrial ISC Assembly Modulating Ferroptosis

ISC constitutes the reactive center and is functionally important for numerous proteins associated with fundamental cellular processes, including the TCA cycle, electron transport chain, DNA damage/repair and oxygen metabolism, due to the chemical and structural versatility [157]. ISC is mainly assembled in the mitochondrial matrix by the conserved mitochondrially localized ISC assembly machinery, which is mainly composed of the dedicated scaffold protein ISCU, the iron donor molecule FXN (Frataxin), the cysteine desulfurase NFS1, and the small partner protein ISD11 [158]. It has been reported that *FXN*, the major causative gene of Friedreich ataxia, is possibly involved in ferroptosis. Friedreich ataxia is a progressive neuro- and cardio-degenerative disorder characterized by ataxia, sensory loss, and hypertrophic cardiomyopathy. Friedreich ataxia patient-derived fibroblasts, as well as the *FXN* mutated murine fibroblasts, are more sensitive to erastin than the normal control cells [159]. Silencing *FXN* in HT-1080 cells represses cell proliferation and induces mitochondrial damage with elevated iron deposition, which leads to the higher ferroptotic sensitivity to erastin. Conversely, ectopic expression of *FXN* maintains mitochondrial integrity and thus mitigates ferroptosis [160]. Similarly, *FXN* deficiency also increases ferroptosis susceptibility in cultured adipocytes. RNAseq analysis indicated ferroptosis as one of the enriched pathways during *FXN* ablation, further arguing the potent role of FXN in ferroptosis [161]. These studies combined with the previous identification that Friedreich ataxia is pathologically manifested by mitochondrial dysfunction, lipid peroxidation, elevated ROS generation, GSH depletion, and increased iron availability, indicate the possibility of pathological engagement of ferroptosis in cell degeneration during the pathogenesis of Friedreich ataxia [159,162]. It is supposed that defective expression of *FXN* (experimental knockout or inherited mutation leading to FXN loss in Friedreich ataxia) leading to a disturbed ISC assembly and thus accumulation of mitochondrial iron would facilitate mitochondrial lipid peroxidation and aggravate ferroptosis. Additionally, defective ISC assembly due to *FXN* deficiency results in loss of ISC binding of IRP1 and robustly activates the cytosolic iron-starvation response. The subsequent upregulation of TFRC and decrease of ferritin thus leads to elevated labile free iron, which further catalyzes lipid peroxidation and navigates ferroptosis (Figure 3). This is verified by the other study reporting that suppression of *NFS1*, the cysteine desulfurase abstracting the sulfur atom from cysteine for ISC assembly, predisposes cancer cells to ferroptosis and slows tumor growth [163]. Cystine/cysteine deprivation could trigger ferroptosis in diverse cells. However, it is still elusive whether disturbed ISC biosynthesis due to the cystine/cysteine deprivation is responsible for this ferroptosis initiation [164]. Moreover, CISDs (CDGSH iron sulfur domain, also known as mitoNEET including CISD1, CISD2 and CISD3) are mitochondria associated iron-containing proteins responsible for the export of ISC [165]. Knockdown of *CISD1* and *CISD2* renders ferroptosis [153,154], further shedding light on the pivotal role of mitochondrial ISC metabolism in modulating ferroptosis. Alternatively, ISC could be also synthesized in cytosol by the CIA (cytosolic iron–sulfur protein assembly) machinery [166]. However, it is still elusive whether CIA is coupled with the ferroptotic sensitivity or not.

### 4.4. Mitochondrial Heme Biosynthesis Modulating Ferroptosis

Heme is other ubiquitous iron-containing molecules important for numerous metabolic enzymes. De novo heme biosynthesis depends on the inter-metabolites shuttling across the mitochondria. Whether heme biosynthesis itself is associated with ferroptosis or not is still unclear. Similar to ISC assembly, it is possible that disturbed heme biosynthesis leads to deposition of mitochondrial iron, which catalyzes mitochondrial lipid peroxidation and initiates ferroptosis. Mitochondrial SFXN2 (sideroflexin 2) is involved in heme biosynthesis with an unclear mechanism. It was reported that *SFXN2* deficient cells possess higher mitochondrial iron accompanied with decreased heme and reduced heme-dependent enzyme activities, and these mutant cells are more sensitive to erastin [167]. After ICH (intracerebral hemorrhage), hemoglobin is released from lysed erythrocytes, then absorbed by microglia and metabolized to heme and free iron, which induce lethal ROS accumulation and are thus neurotoxic [168]. Several types of cell death have been identified for neuronal death during ICH in human patients and experimental animals, including apoptosis and necrosis. It was reported that ferroptosis is also involved in this ICH injury. Ferrostatin-1 significantly reduces hemoglobin induced lipid ROS accumulation, GPX activity deficit and neuronal death in organotypic hippocampal slice cultures in vitro. Moreover, ferrostatin-1 also alleviates neuronal degeneration, iron deposition, injury volume, and neurologic deficit after ICH in vivo, further indicating an involvement of hemoglobin/heme in ferroptosis [169]. Additionally, heme produced from hemoglobin degradation also induces ferroptosis in anucleate platelets [170] and in HT-1080 fibrosarcoma cells [62]. It is still unclear whether heme itself or its catabolite iron is responsible for this ferroptosis regulation. HO-1 catabolically degrades heme and generates carbon monoxide, biliverdin, and iron [171]. It has been reported that the expression of *HO-1* aggravates ferroptosis, which is possibly due to the iron release via heme catabolism. Pharmacological inhibition or genetic ablation of *HO-1* overtly ameliorates ferroptosis [62,64,172]. However, the opposite conclusion was drawn by independent studies. Depletion of *HO-1* increases the sensitivity of renal proximal tubule cells to erastin induced ferroptosis [173]. Similarly, Sun and colleagues showed that knockdown of *HO-1* by RNAi facilitates ferroptosis induced by erastin and the system X_C_^-^ inhibitor sorafenib in hepatocellular carcinoma cells [65]. This is possibly due to the catabolite biliverdin, which possesses reducing properties and is recognized as potent antioxidants [174]. Altogether, mitochondria act as the center for cellular iron metabolism and dominate both mitochondrial and the whole cellular iron status, thus determining the ferroptotic sensitivity.

### 4.5. Mitochondrial Glutaminolysis-TCA-ETC Axis Modulating Ferroptosis

Glutaminolysis-TCA cycle with the coupled ETC-ATP production in mitochondria was characterized as one of the most important metabolisms involved in the regulation of ferroptosis. Glutamine is the most abundant amino acid and serves as the nitrogen source for the anabolism of nucleotides, amino acids, and hexamine, as well as acts as the carbon source to fuel TCA cycle through glutaminolysis [175]. Multiple cancer cells are addicted to high concentration of glutamine for the growth and proliferation in culture [176,177]. An earlier study reported that transporter SLC38A1 essential for glutamine uptake is a positive regulator of ferroptosis by using a pooled shRNA lentivirus library screening assay [60]. Moreover, miR-137 suppresses ferroptosis by targeting glutamine importer SLC1A5. Similarly, chemical inhibition of SLC1A5/SLC38A1 by the inhibitor GPNA (L-g-glutamyl-p-nitroanilide) sharply reduces cytotoxicity of erastin in melanoma cells [178]. Furthermore, mitochondrial glutaminase GLS including GLS1 and GLS2 are responsible for the glutaminolysis to catabolize glutamine and yield glutamate. Knockdown of *GLS2*, but not *GLS1*, or chemical inhibition of GLS by Compound 968, significantly decelerates ferroptotic cell death. Glutamic-oxaloacetic transaminase GOT1 is subsequently responsible for the conversion of glutamate into α-KG (α-ketoglutarate), which further fuels TCA cycle. *GOT1* RNAi or pharmacological inhibition of the transaminases activity by AOA (amino-oxyacetate) inhibits the execution of ferroptosis, while this ferroptosis could be restored by supplementation of α-KG [115,178,179,180]. Similarly, miR-9 could reduce erastin and RSL3 induced ferroptosis by targeting GOT1 [181]. It was also elaborated that glutaminolysis product α-KG and its downstream TCA metabolites including succinate, fumarate, and malate are all intrinsic metabolites which could modulate cysteine-deprivation induced ferroptosis [116]. It is supposed that glutaminolysis-TCA cycle supported electron transport may facilitate lipid peroxidation through generating ROS, which is produced as the inevitable by-product of mitochondrial respiration. Alternatively, the glutaminolysis-TCA cycle and the coupled ETC-ATP production could modulate AMPK activity and thus determine ferroptotic sensitivity [182]. More specifically, AMPK activation phosphorylates and thus inhibits acetyl-CoA carboxylase 1 and 2 (ACC1/2), leading to a reduced conversion of acetyl-CoA to malonyl-CoA required for the biosynthesis PUFAs [183], which in turn protects cells from lipid peroxidation and ferroptosis [183,184]. Moreover, AMPK activation leads to downregulation of SREBP (sterol regulatory element-binding protein 1) and its downstream SCD1 (stearoyl-coenzyme A (CoA) desaturase-1), the primary enzyme for the biosynthesis of anti-ferroptotic MUFAs [185]. Altogether, these studies thus highlight a critical role of mitochondrial metabolism in regulation of lipid peroxidation and ferroptosis via the metabolic process including glutaminolysis-TCA cycle-ETC-ATP production (Figure 3).

### 4.6. Mitochondrial NADPH Modulating Ferroptosis

NADPH is always used as an essential electron donor in several redox reaction catalyzed by some enzymes. It is recognized that NADPH functions importantly in fine-tuning ferroptosis. Tonnus and Linkermann hypothesized that NADPH gradient defines the ferroptotic risk and directs the progression of synchronized regulated necrosis of renal tubules cells in ischemia-reperfusion injury [186,187]. By using a compound screening with high cell-line-selective lethality, NADPH was identified as a biomarker for ferroptotic sensitivity across multiple cell lines [188]. Mechanistically, it seems complicated for NADPH to antagonize ferroptosis. NADPH could support GSH reductase to convert GSSG and yield reduced GSH [189,190]. Furthermore, NADPH assists ferroptosis regulator FSP1 for the reduction of CoQ10 to trap lipophilic radical and halts the propagation of lipid peroxides and suppresses ferroptosis [34,35]. Although the pentose phosphate pathway, a metabolic branch from glycolysis, is the major metabolic process responsible to produce NADPH [191], mitochondria are one of the major compartments for NADPH metabolism. NADPH could be generated by the oxidation of malate to pyruvate by malic enzyme inside mitochondria [192]. Alternatively, mitochondrial IDH2 (isocitrate dehydrogenase 2) could catalyze the oxidative decarboxylation of isocitrate to α-KG by reducing NADP^+^ to NADPH [193]. NADPH could also be generated by cytosolic NADP^+^-dependent dehydrogenases (including isocitrate dehydrogenase 1 and malic enzyme) utilizing mitochondrial TCA intermediates isocitrate and malate as metabolic precursors [194]. Knockdown of *IDH2* substantially increases the susceptibility of ferroptosis in cultured tumor cells and in allografted Hepa1–6 tumor in nude mice [195]. Although the extra-mitochondrial NADPH (mainly plasma membrane) was evidenced as the major lipophilic radical-trapping antioxidant to prevent lipid damage and counteract ferroptosis, these studies put forward the possibility that mitochondria may modulate ferroptosis through NADPH metabolism.

### 4.7. Other Mitochondrial Components and Ferroptosis

Besides, there are other research elaborating additional mitochondria associated molecules in determining ferroptosis (Figure 3). The shRNA screening by using the library targeting mitochondrial genes identified that *RPL8* (ribosomal protein L8), *ATP5G3* (ATP synthase F0 complex subunit C3), *CS* (citrate synthase), *TTC35* (tetratricopeptide repeat domain 35), and *ACSF2* (acyl-CoA synthetase family member 2) are required for erastin induced ferroptosis. Among them, ACSF2 and CS are implicated in mitochondrial fatty acid metabolism, indicating that lipid synthesis may supply a specific lipid precursor required for ferroptosis [3]. Mutation of mitochondrial TCA cycle enzyme FH (fumarate hydratase) leads to ferroptotic resistance, further suggesting an involvement of mitochondrial TCA cycle in ferroptosis modulation, as discussed above [116]. Furthermore, the mitochondrial protease LONP1 (Lon peptidase 1) [196] and the pro-apoptotic BID [110] are also regulators for ferroptosis under the corresponding contexts, although the underlying mechanism is unclear. Many canonical ferroptosis proteins including ASCL4 and P53 possess partially mitochondrial localization. However, it is still elusive whether this portion has any potential role in the regulation of ferroptosis.

## 5. Conclusive Remarks and Perspective

Ferroptosis is a newly characterized form of RCD, which is typically manifested by the GSH exhaustion, GPX4 inactivation, and lethal accumulation of lipid peroxidation. It has been widely recognized that dysregulated ferroptosis accounts, at least partially, for the cell degeneration and tissue damage during the pathogenesis of diverse human diseases, especially the neurodegenerative diseases and organ injury related diseases (Table 1). Targeted induction of ferroptosis is considered as a highly potential therapeutic approach for the clinical intervention of other therapy-resistant cancers.

As the powerhouse for ATP production, mitochondria also provide the proper compartment for series of cellular metabolisms. Besides, mitochondria act as a signaling organelle for diverse signaling pathways in response to intracellular and extracellular clues. It has been extensively evidenced that mitochondria determine several types of RCDs. Although some studies proposed that other organelles are involved in ferroptosis, including endoplasmic reticulum [22], Golgi [197] and lysosome [198], increasing studies as mentioned above have put forward the possibility of multifaceted regulation of ferroptosis by mitochondria. Mitochondria are compromised as evidenced by the abnormal morphology, burst of mitochondrial ROS, alteration of mitochondrial membrane potential, elevated mitochondrial lipid peroxidation, as well as accumulation of mitochondrial iron during ferroptosis in different models. More importantly, emerging evidence has been presented to elaborate a multifaceted regulation of ferroptosis by mitochondria associated metabolisms and molecules, although some controversies still exist. It is worth noting that both mitochondrial compromise and typical ferroptosis characteristics are manifested in diseased tissues of patients with neurodegenerative diseases or organ injury related diseases. For example, mitochondrial dysfunction has been widely recognized as an early event during the pathogenesis of Alzheimer’s disease, including elevated mitochondrial oxidative damage and abnormal mitochondrial morphology due to the imbalanced mitochondrial fission and fusion [199]. The typical hallmarks of ferroptosis including iron deposition, depleted GSH and elevated lipid peroxidation, were also observed during the pathogenesis of Alzheimer’s disease [200,201,202]. Besides, both mitochondria-targeting therapeutic approaches and iron-targeting therapeutic strategies have been developed [203,204]. More extensive investigations are needed to further dissect the relevance of mitochondrial dysfunction and ferroptosis in the pathogenesis of Alzheimer’s disease and other related diseases. It is thus of potential treatment efficacy by combination of ferroptosis-targeting and mitochondria-targeting therapeutic approaches for the clinical treatment of these diseases.

**Table 1:** Human diseases linked to ferroptosis.

Disease	Model	Ferroptosis Related Index	Ferroptosis Related Reagents	References
Intracerebral hemorrhage	1. Rat intracerebral injection of autologous whole blood 2. Murine models with striatum injection of collagenase	GPX4↓ GSH↓ ROS↑	Iron chelator: VK-28 Inhibitor: Ferrostatin-1	[205,206,207]
Cardiomyopathy	1. Murine models of doxorubicin-induced cardiomyopathy 2. Murine models of ischemia/reperfusion-induced cardiomyopathy	GSH↓ GPX4↓ *Ptgs2*↑ 4-HNE↑	Iron chelators: DFO, dexrazoxane Inhibitor: Ferrostatin-1	[64,115]
Atherosclerosis	Iron-loaded *ApoE*-/-*FPN*^wt/C326S^ mice	GPX4↓ GSH↓ ROS↑ MDA↑ HO-1↑ 4-HNE↑	Iron chelator: DFO	[208,209]
Nonalcoholic fatty liver disease	Mice fed by the CDAA-based high-fat diet	GSH↓ MDA↑	Iron chelator: DFP	[210,211]
Acute Kidney Injury	1. Rats renal ischemia/reperfusion 2. Mice models with lipopolysaccharide injection 3. Mice models with BSO injection 4. *GPX4* knockout mice	GPX4↓ GSH↓ HO-1 ↑ MDA↑ 4-HNE↑ ROS↑	Inducer: RSL3 Inhibitor: Liproxstatin-1, Ferrostatin-1 Iron chelators: DFO	[212,213,214]
Chronic obstructive pulmonary disease	1. Mice models exposed to whole body mainstream cigarette smoke 2. *GPX4* knockout mice and transgenic mice	GPX4↓ GSH↓ iron↑ Transferrin↑ MDA↑ 4-HNE↑	Iron chelator: DFO Inhibiter: Ferrostatin-1	[215,216,217]

Abbreviations: ApoE, apolipoprotein E; BSO, buthionine sulfoximine; CDAA, choline-deficient L-amino acid defined diet; DFO, deferoxamine; DFP, deferiprone; FPN, ferroportin; GPX4, glutathione peroxidase 4; GSH, glutathione; 4-HNE: 4-hydroxynonenal; HO-1: heme oxygenase 1; MDA: malondialdehyde; *Ptgs2*: prostaglandin-endoperoxide synthase 2; ROS, reactive oxygen species.

## Data Availability

Not applicable.
